# Identification and characterization of endonuclein binding proteins: evidence of modulatory effects on signal transduction and chaperone activity

**DOI:** 10.1186/1471-2091-10-34

**Published:** 2009-12-22

**Authors:** Maja Ludvigsen, Morten Østergaard, Henrik Vorum, Christian Jacobsen, Bent Honoré

**Affiliations:** 1 Department of Medical Biochemistry, Aarhus University, Ole Worms Allé 3, Building 1170, Aarhus, DK-8000 Aarhus C, Denmark; 2 Department of Ophthalmology, Aalborg Hospital, Aarhus University Hospital, Hobrovej 10, DK-9000 Aalborg, Denmark

## Abstract

**Background:**

We have previously identified endonuclein as a cell cycle regulated WD-repeat protein that is up-regulated in adenocarcinoma of the pancreas. Now, we aim to investigate its biomedical functions.

**Results:**

Using the cDNA encoding human endonuclein, we have expressed and purified the recombinant protein from *Escherichia coli *using metal affinity chromatography. The recombinant protein was immobilized to a column and by affinity chromatography several interacting proteins were purified from several litres of placenta tissue extract. After chromatography the eluted proteins were further separated by two-dimensional gel electrophoresis and identified by tandem mass spectrometry. The interacting proteins were identified as; Tax interaction protein 1 (TIP-1), Aα fibrinogen transcription factor (P16/SSBP1), immunoglobulin heavy chain binding protein (BiP), human ER-associated DNAJ (HEDJ/DNAJB11), endonuclein interaction protein 8 (EIP-8), and pregnancy specific β-1 glycoproteins (PSGs). Surface plasmon resonance analysis and confocal fluorescence microscopy were used to further characterize the interactions.

**Conclusions:**

Our results demonstrate that endonuclein interacts with several proteins indicating a broad function including signal transduction and chaperone activity.

## Background

Pancreatic cancer is the seventh most common cause of cancer death in Western society with a 5-year survival rate of approximately 5.5% being the lowest 5-year relative survival rate of all cancers. The incidence rate of this aggressive tumor is increasing and the treatment is ineffective with a poor prognosis [1,2]. Recently, gathering of data concerning genomics and proteomics studies of pancreatic cancer showed differential regulation of 2,516 potential biomarkers [3]. These include known oncogenes and cell cycle regulators, *e.g.*, the *ras *oncogene [4], p53 [5], SMAD4 [6], epidermal growth factor and epidermal growth factor receptor [7], cyclin D1 [8], CDK4I [9], as well as insulin-like growth factor-1 receptor, stat-3 and the tyrosine kinase *src *[10]. Still, the molecular biology of the disease is not well understood and a more thorough investigation of the specific proteins is crucial

In an earlier study, we have revealed that endonuclein [11], a cell cycle regulated WD-repeat protein, also contributes to the development of the protein phenotype of pancreatic cancer. WD-repeat proteins usually consist of 4-16 WD-repeats forming an up to eight blade propeller structure creating a stable platform that may form complexes reversibly with several proteins [12]. Despite of the structural similarity between these members they are a functionally diversified group where all members seem to possess regulatory functions *e.g*. proteins including cell division control proteins, coatomer b subunits, transcription initiation factors, microtube associate proteins, actin interacting proteins, as well as enzymes like protein phosphatase 2A and myosin heavy chain kinase A 3-13 [13-23]. To date, the function of the various protein members have thus been ascribed to crucial regulatory roles in signal transduction, RNA processing, gene expression, trafficking of vesicles, assembly of cytoskeletal components as well as participation in the regulation of the cell cycle [24].

Since several WD-repeat proteins are known to form multiprotein complexes by interacting with other proteins sometimes through their WD-repeat domains, we set forward studies to reveal which function(s) that may be attached to endonuclein by identifying interacting proteins. Consequently, we have expressed recombinant endonuclein in *Escherichia coli *and used the purified immobilized protein to affinity purify several endonuclein interacting proteins from a placental tissue extract. A proteomic approach using two-dimensional gel electrophoresis (2D-PAGE) and tandem mass sepctrometry (MS/MS) [25] together with a number of proteins assays were used to further characterize the proteins. The nature of the affinity purified proteins confirms that endonuclein may possess regulatory functions. Thus, among the interacting proteins, we have identified, are TIP-1 (a PDZ protein that interacts with the oncogenic HTLV-1 Tax protein), mt-SSB (a transcription factor) and a number of stress related proteins belonging to the molecular chaperones. Thus, we have discovered and characterized a number of putative functions of endonuclein that will be discussed in light of the disclosure of the interacting partners.

## Results

**Figure 1:**
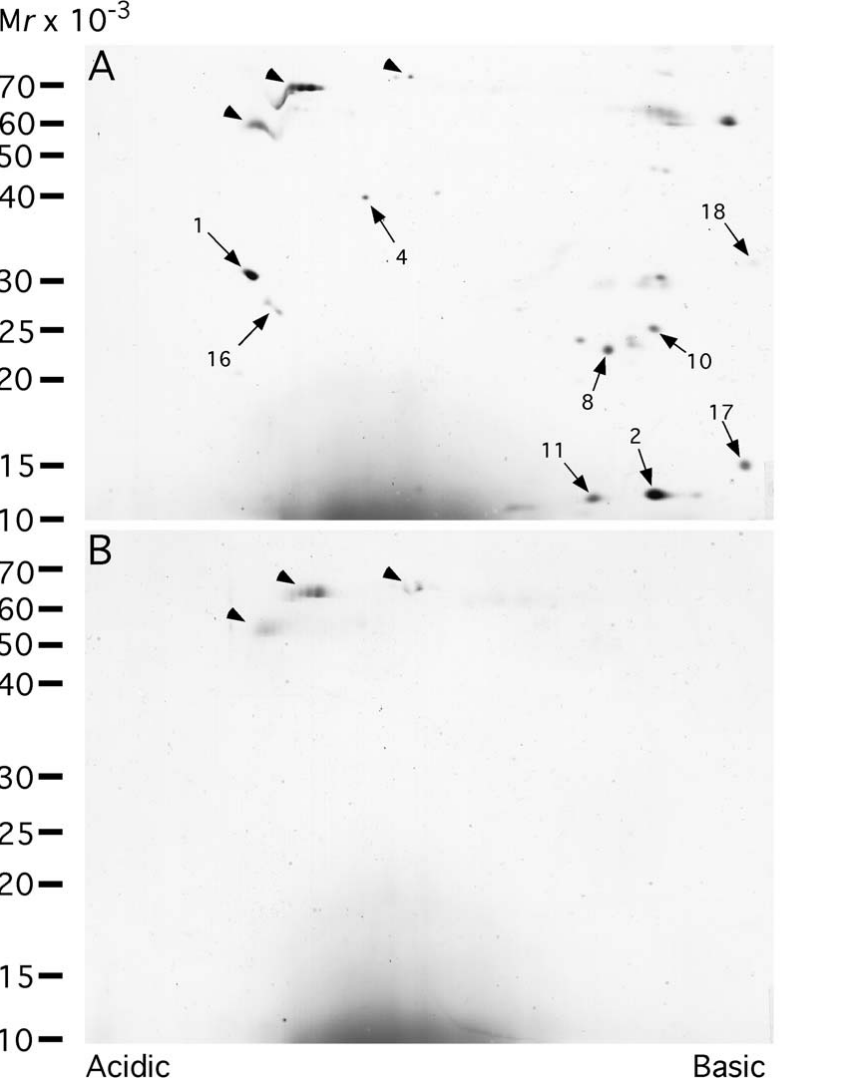
**Affinity purification of endonuclein interacting proteins**. A placental tissue extract was applied to a column with immobilized endonuclein, (A), and to a parallel column without endonuclein, (B), in a physiologic buffer. Proteins were eluted from both columns at pH 2.7. The eluates were neutralized and finally subjected to two-dimensional gel electrophoresis with subsequent silver staining. Arrows with numbers indicate protein spots that were excised from the gel and subjected to nano-electrospray tandem mass spectrometry for identification. The partial sequences and the names of the identified proteins are shown in Table 1. Proteins that interacted unspecifically with the column matrix are given with arrowheads.

In order to detect endonuclein interacting proteins, we immobilized recombinant endonuclein to a column and prepared a reference column without immobilized endonuclein. Similar volumes of placental tissue extract were added to both columns at neutral pH. After a wash of both columns with 1 M NaCl, proteins that interacted with the immobilized endonuclein were eluted with 0.1 M glycine buffer (pH 2.7) and subsequently neutralized with 1 M Tris-HCl, pH 9. Both eluates from the endonuclein column and the reference column were concentrated and subjected to 2D-PAGE as shown in Fig 1. Several proteins were found to interact specifically with endonuclein as indicated with numbers in Fig 1A. A few proteins interacted non-specifically with the column matrix as given with arrowheads in Fig 1B. Of several proteins that were subjected to partial internal sequencing by MS/MS, eight spots could be unambiguously identified as given in Table 1. The localization of the identified interacting proteins is in line with the previously established nuclear localization and cytoplasmic localization in the endoplasmic reticulum of endonuclein by immunoelectron and confocal laser scanning microscopy [11]. Each of the proteins will be described in detail below.

**Table 1:** Identification of endonuclein interacting proteins by peptide sequencing using tandem mass spectrometry.

Protein spot No.	Peptide sequence obtained by tandem mass spectrometry	Identification of protein	Localization	Mr (kDa)	pI
1	ELEEIVQPIISK	BiP/Grp78	ER resident	72.1	4.9

2	VSEGGPAEIAGLQIGDK	TIP-1	Cytoplasm* Nucleus	12.9	9.2

4	TLEVEIEPGVR	DNAJB11/HEDJ (EIP-4)	ER/secreted	40.5	5.8

8	TGAELVTCGSVLK	SDF2-like protein 1 (EIP-8)	ER resident	23.6	6.6

10	Failed				

11	VSEGGPAEIAGLQIGDK	TIP-1	Cytoplasm* Nucleus	12.9	9.2

16	SQIFSTASDNQPTVTIK TFDLTGI LTPEEIER NELESYAYSLK KKELEEIVQPIISK	BiP/Grp78	ER resident	72.1	4.9

17	SGDSEVYQLGDVSQK VGQDPVLR	Mt-SSB (ssDNA binding protein/P16)	Mitochondria Nucleus* Cytoplasm	17.2	10.5

18	ENKDVLTFTCEPK ILILPSVTR LSIPQITTK LFIPQITTK	Pregnancy specific β-1 glycoproteins (PSBGs)	ER/secreted	36.2-52.7	8.0-9.3

*Determined by laser scanning confocal microscopy in present work

### Identification of protein spots No. 1 and No. 16

**Figure 2:**
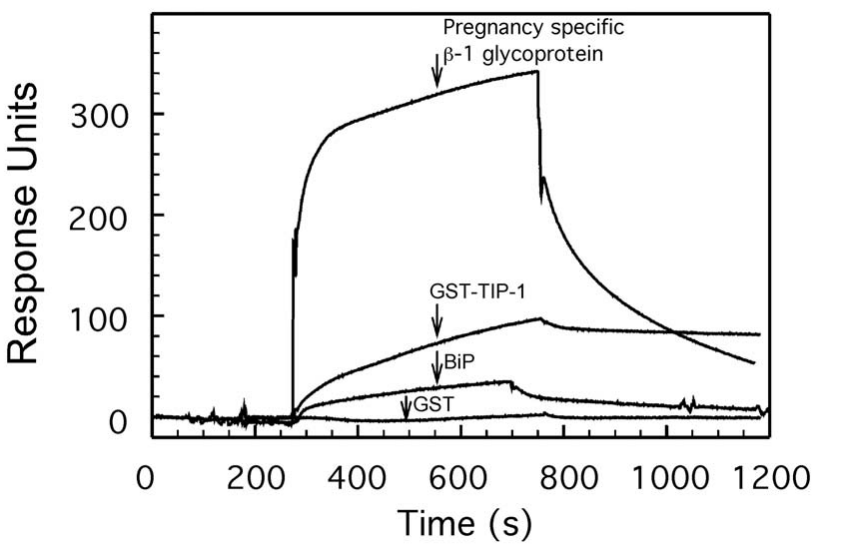
**Surface plasmon resonance analysis of the binding of proteins to endonuclein**. Endonuclein was immobilized to a sensor chip and the on and off rates for ligand binding were recorded on a BIAcore 2000. The recorded sensorgrams show binding of 1 μM of pregnancy specific β-1 glycoprotein, 5 μM recombinant TIP-1 as fusion peptide with glutathion S-transferase (GST-TIP-1), 1 μM BiP and 1 μM of pure glutathion S-transferase (GST) as a control. Interaction with endonuclein was verified with all proteins except GST which revealed no detectable binding.

Protein spot No. 1 gave the peptide sequence ELEEIVQPIISK which was identified as a peptide located in the C-terminal of immunoglobulin heavy chain binding protein BiP or Grp78 [26]. Similarly, protein No. 16 gave 5 peptides (Table 1) that also were found in BiP. Peptide spots No. 1 and 16 with molecular masses around 25-30 kDa thus represent C-terminal cleavage products of BiP that has a molecular mass around 72 kDa. In order to verify the interaction with BiP, we performed surface plasmon resonance experiments by flowing hamster BiP that possesses more than 98% sequence identity to human BiP over the immobilized endonuclein. As seen from Fig 2 these experiments verified that there was an interaction between the immobilized endonuclein and BiP. As a control recombinant GST revealed no detectable binding.

### Identification of protein spots No. 2 and No. 11

**Figure 3:**
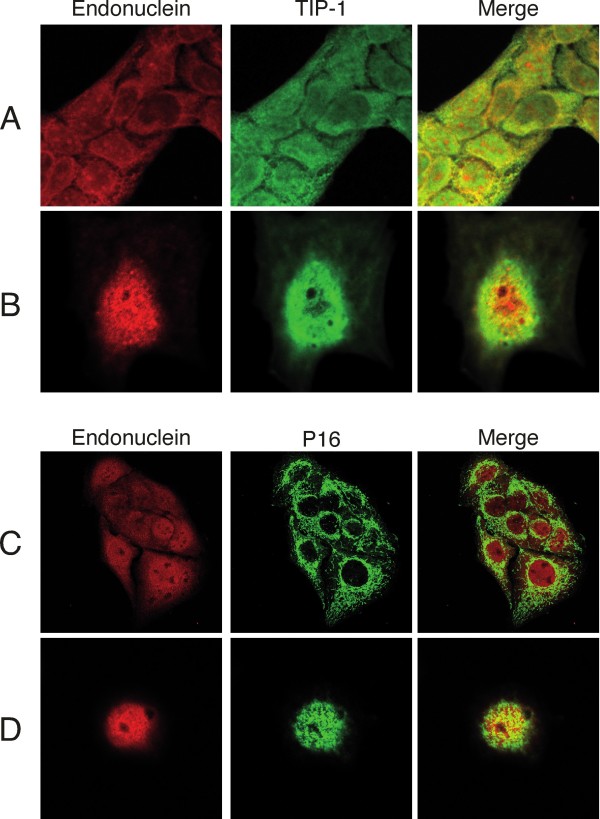
**Immunocytochemistry stainings of HaCat keratinocytes**. (A) Double staining of endonuclein and TIP-1 showing co-localization in the cytoplasm (x40) and (B) in the nucleus (x100). (C) Double staining of endonuclein and mt-SSB showing no co-localization (x40) and (D) co-localization in the nucleus (x63).

Both spot No. 2 and No. 11 gave the sequence VSEGGPAEIAGLQIGDK that identified the protein as Tax interaction protein 1, TIP-1 [27,28]. The localization of protein spots No. 2 and No. 11 in the 2D gel is in agreement with the deduced molecular mass of 12.9 kDa and pI of 9.2 for TIP-1. The surface plasmon resonance experiments shown in Fig 2 were used to verify that recombinant TIP-1 protein constructed as fusion protein with GST interacted with endonuclein. Separate control experiments revealed that pure GST protein did not interact with endonuclein. To establish evidence that endonuclein and TIP-1 are localized to the same subcellular compartment, we performed double immunocytochemistry on cultured HaCat cells. As shown in Fig 3 the two proteins co-localize in the cytoplasm (Fig 3A) and to some extend in the nucleus (Fig 3B).

### Identification of protein spot No. 4

**Figure 4:**
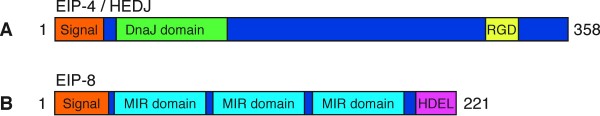
**Structure of two endonuclein interacting proteins**. (A) Endonuclein interacting protein 4, DNAJB11, contains a signal sequence that is putatively cleaved off between Ala-22 and Gly-23. It contains a DnaJ domain (green box) and an RGD sequence (yellow box) and might be secreted due to the lack of an ER retrieval sequence. (B) SDF2-like protein 1 contains a signal sequence that is putatively cleaved off between Ala-28 and Ala-29. It contains three MIR domains (blue box) and a HDEL ER retrieval sequence (purple box).

Protein spot No. 4 revealed the peptide sequence TLEVEIEPGVR that identified the protein as DnaJ homolog subfamily B member 11, DNAJB11 [29]. The molecular mass of the predicted protein is calculated to be 40.5 kDa and the pI to 5.8, *i.e*. very close to the experimental values observed, see Fig 1. By using the publicly available WWW server that is based on neuronal networks [30,31], the N-terminal 22 amino acids were found to have the hydrophobic characteristics of a signal peptide for directing the protein into the ER with a likely cleavage site between Ala-22 and Gly-23 which is consistent with the protein localization in strong association with the ER membrane oriented towards the ER lumen [29]. The protein also exhibits a J domain after the signal sequence. The J domain is a highly conserved domain of about 70 amino acids found in the hsp40 protein family that consist of over 100 members [32]. Further towards the C-terminal there is an RGD motif which may serve a role in cell adhesion, since this sequence can bind to the integrins [33]. The different domains of DNAJB11 are shown in Fig 4A.

### Identification of protein spot No. 8

Protein spot No. 8 gave the sequence TGAELVTCGSVLK that identified the protein as stromal cell-derived factor 2-like protein 1, SDF2-like protein 1 [34]. By analyzing the sequence using the WWW server [30,31], we found a putative signal sequence with an indicated cleavage site between Ala-28 and Ala-29. Furthermore, the protein terminates with the HDEL signal which has been identified as an ER retrieval signal in some of the CREC proteins [35-37] suggesting that the protein is localized as a resident protein within the ER. The database search showed that the protein possesses a strong similarity to the stromal cell derived factor-2, SDF-2 [38] which has been reported to contain MIR domains, since these are also found in protein *O*-mannosyl-transferases, inositol 1,4,5-trisphosphate receptors and ryanodine receptors [39]. The three MIR domains present in SDF2-like protein 1 are shown in Fig 4B.

### Identification of protein spot No. 17

From spot No. 17, we obtained two peptide sequences; SGDSEVYQLGDVSQK and VGQDPVLR. Both peptides showed a perfect identity match to the mitochondrial single-stranded DNA binding protein, mt-SSB [40]. To further investigate this interaction, we performed double immunocytochemistry on cultured HaCat cells (Fig 3). In most cells, endonuclein and mt-SSB did not co-localize to the same cellular compartment (Fig 3C). However, few cells showed a clear presence of mt-SSB in the nucleus co-localizing with endonuclein (Fig 3D). This is consistent with the fact, that this protein has been reported to be mainly localized in the mitochondria, while small amounts have been detected within the nucleus and in the cytoplasm [41]. In addition, it has been shown that mt-SSB is a transcription factor for the Aα fibrinogen gene [42]. The mt-SSB gene has been assigned to chromosome 7 at 7q34 [43], spanning about 12 kb.

### Identification of protein spot No. 18

Spot No. 18 revealed the peptides ENKDVLTFTCEPK, ILILPSVTR, LSIPQITTK and LFIPQITTK. By searching the databases, we found that these peptides corresponded to the pregnancy specific β-1 glycoprotein 1 (PSBG-1) branch of the carcinoembryonic antigen family, where to date 11 proteins have been identified, PSBG1 - PSBG11 [44]. The PSGs are abundantly expressed in placenta during embryonic development and also in uterus, pancreas, testis and fetal liver. The PSGs contain signal sequences and are secreted proteins [44]. Many of them contain an RGD sequence which may serve a role in cell adhesion, since this sequence may bind to the integrins [33]. The peptides identified (Table 1) may correspond to several of the PSBGs. Surface plasmon resonance experiments with a commercial preparation of PSBG indeed verified that endonuclein may interact with members of this class of proteins (Fig 2).

### Ca^2+^-binding to endonuclein

**Figure 5:**
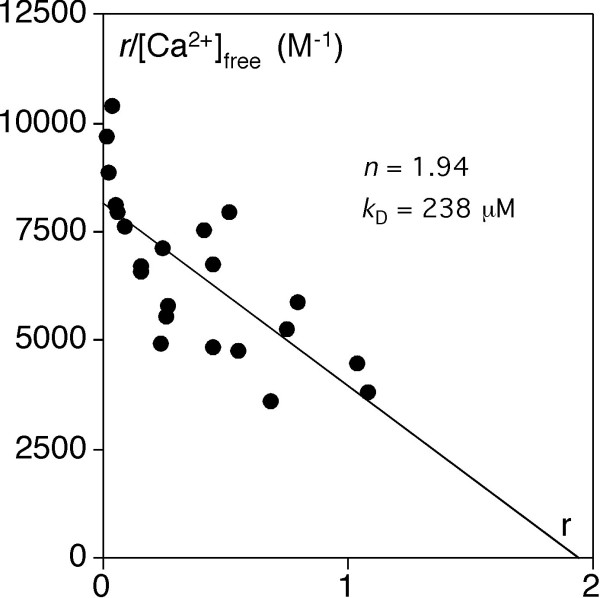
**Ca^2+^-binding to endonuclein**. Binding of Ca^2+ ^was measured in 150 mM KCl, 50 mM Hepes, pH 7.4 at 37°C with the rate dialysis procedure [45]. The data were fitted to the Scatchard equation and the analysis was in agreement with a model consisting of two independent sites (*n* = 1.94) each with the dissociation constant *k*_D _= 238 μM.

Binding of Ca^2+ ^to endonuclein was measured with the protein in solution using the rate dialysis technique [45]. A Scatchard plot of the data is shown in Fig 5. The data obtained may be interpreted by a model where endonuclein contains two Ca^2+^-binding sites, each binding Ca^2+ ^with a dissociation constant, *k*_D_, of 240 μM at physiologic conditions.

## Discussion

Endonuclein, previously known as IEF SSP 9502 [46] and the yeast homologue PWP1, are WD-repeat proteins that to date have been described in a very limited number of papers [11,46-51]. In previous studies endonuclein has been reported to be up-regulated at the transcript level [47] as well as at the protein level [11] in adenocarcinoma of the pancreas. Up-regulation of this protein in cancer may be in line with the fact that endonuclein is identified as a cell cycle regulated protein synthesized at increasing levels and with nuclear focusing during interphase [11]. Up-regulation of the protein may promote the cell cycle in line with the observation that yeast cells with a null mutation of the homologous gene (PWP1) grow slowly [50]. PWP1 is involved in ribosome biosynthesis through its interaction with *trans*-acting ribosome biogenesis factors and with several ribosome protein subunits [51]. In addition, it has recently been shown that PWP1 associates with the 25S ribosomal DNA chromatin (rDNA), and that PWP1 may play a role in regulation of rDNA transcription [48]. Furthermore, the recent finding that endonuclein is downregulated during heat stress-induced apoptosis [49], suggests that indeed this protein is involved in diverse important cellular functions. Since WD-repeat proteins as a rule possess regulatory functions rather than enzymatic [24], we further explored the functional roles of endonuclein by biochemical analyses with emphasis on the identification and characterization of its interacting proteins.

**Figure 6:**
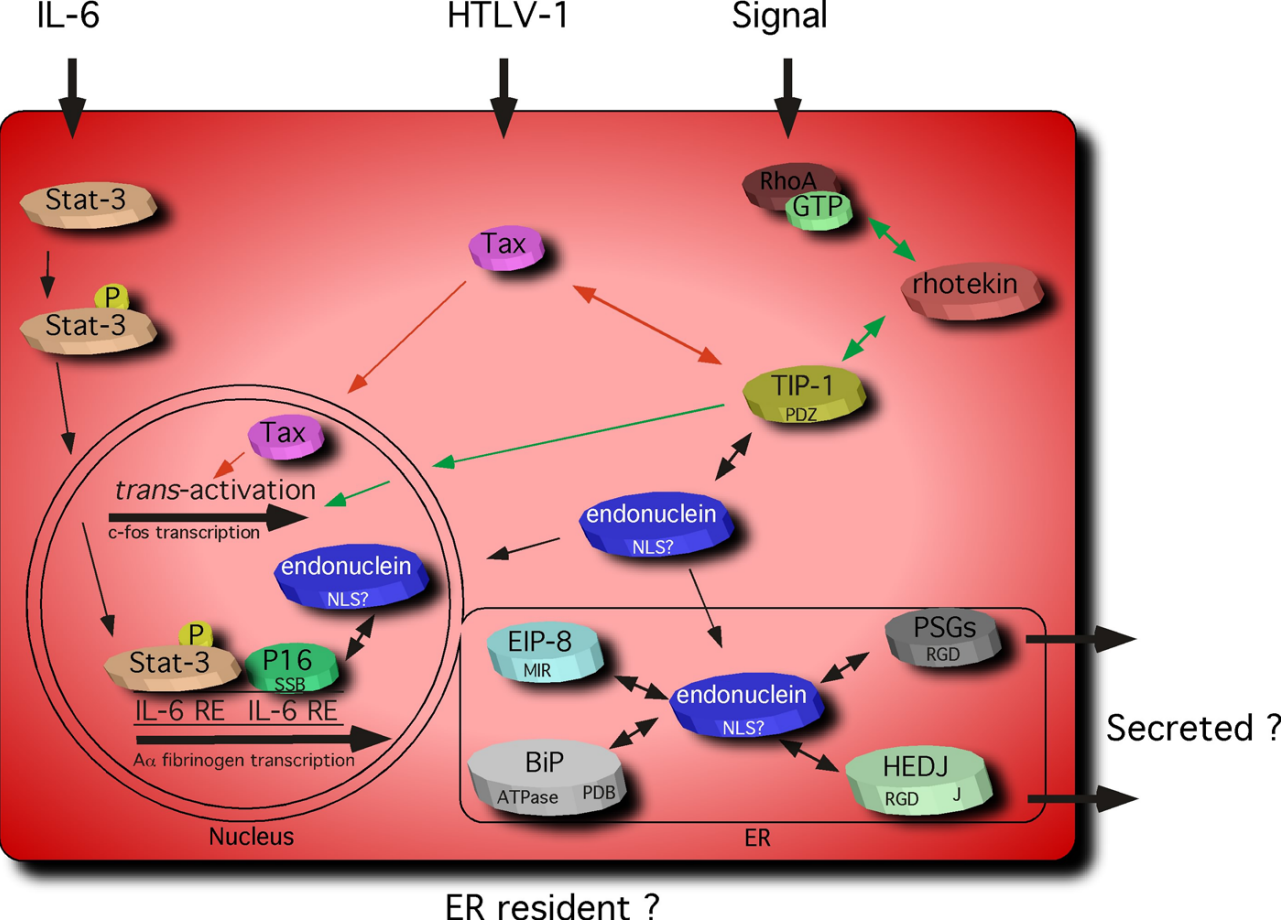
**Model of endonuclein functions**. Endonuclein may modulate several signal transduction pathways to the nucleus as well as chaperone activities in the ER. Through interaction with TIP-1, endonuclein may interfere with the Rho signaling pathway and the pathway leading to oncogenesis by Tax. RhoA, rhotekin and TIP-1 may form a ternary complex in the cytosol (indicated with two-way green arrows) that produces a signal in the nucleus increasing c-fos transcription (indicated with one-way green arrows). Tax may* trans*-activate a number of genes, *e.g*. c-fos (red arrows) through interaction with a number of cellular proteins. IL-6 stimulation activates two signaling pathways one involving the Janus kinases (Jaks) and one involving the MAP kinase cascade. The Jaks phosphorylate stat-3, which is then translocated into the nucleus. Usually, stat-3 then binds to the IL-6 response element (IL-6 RE), however, this does not occur at the Aα fibrinogen promoter that instead binds the transcription factor P16 (mt-SSB). The IL-6 induced transcription of Aα fibrinogen may thus be modulated by interaction of endonuclein with P16. In the ER endonuclein may modulate chaperone activities by interacting with the membrane bound co-chaperone HEJD (DNAJB11) and the chaperone BiP. The significance of the interaction of endonuclein with EIP-8 (SDF2-like protein 1) and PSGs (PSBGs) in the ER is unknown at present. Two-way arrows indicate putative protein-protein interactions. Protein motifs or domains are indicated on the proteins, MIR, RGD, PDB, J, PDZ, SSB and NLS.

Endonuclein interacting proteins (Table 1) may largely be classified into two groups, one that is involved in signal transduction from the cytosol to the nucleus and another that participates in chaperone activities in the ER. These localizations are in line with the previous observation by immunoelectron and laser scanning fluorescence microscopy [11] that endonuclein localizes to the nucleus as well as to the cytoplasm in the endoplasmic reticulum indicating that the interactions observed may be of functional significance. The results are summarized in Fig 6. In the cytosol endonuclein may interact with the Tax interaction protein 1, TIP-1. TIP-1 was originally identified as a PDZ domain protein interacting with the C-terminal of Tax, a 40 kDa nuclear phosphoprotein encoded by HTLV-1 [27], and recent studies have identified TIP-1 as an atypical PDZ protein that functions as a negative regulator of PDZ-based scaffolding [52]. Tax is considered to be oncogenic by *trans*-activating a number of viral and cellular genes [53], and it has been shown that overexpression of TIP-1 reduced the proliferation and growth of colorectal cancer cells [54]. Since Tax is not a DNA binding protein *per se*, it executes its action through the binding of other cellular proteins. However, which specific *trans*-activation pathway that is essential for transformation of cells still remains controversial [53]. Interestingly, TIP-1 that contains a PDZ domain has been reported to interact with the C-terminal of rhotekin [28]. Rhotekin was identified as a target protein of the Rho family of small GTPases [55]. A ternary complex of active Rho with rhotekin and TIP-1 produces a signal in the cytosol that triggers strong activation of the c-fos serum response element [28] that also is activated by Tax [53]. How the signal from the ternary complex in the cytosol is transferred to the nucleus is unknown. The interaction of endonuclein with TIP-1 could thus have modulatory actions on the Rho signal transduction pathway as well as on the pathway that leads to oncogenesis induced by Tax. Such a modulatory effect on signal transduction pathways is further substantiated by the observation that endonuclein may interact with mt-SSB, a transcription factor for the Aα fibrinogen gene [42]. This gene is induced by interleukin-6 (IL-6) which activates two signal transduction pathways; the Janus kinases that phosphorylate a group of latent cytoplasmic transcription factors known as stats and the MAP kinase signaling cascade that activates the C/EBP transcription factors. Upon phosphorylation, stat-3 is translocated to the nucleus and generally binds to the IL-6 response element (IL-6 RE) of the genes that it activates. However, stat-3 does not associate with the IL-6 RE of the fibrinogen promoter. Instead mt-SSB is attached to this site. Upon IL-6 stimulation mt-SSB transiently leaves the site and re-associates again later. The further details of the molecular mechanism whereby IL-6 *trans*-activates the Aα fibrinogen gene remains to be investigated [56,57]. Endonuclein may thus putatively modulate the signal transduction pathway through its interaction with mt-SSB.

In the ER, endonuclein may interact with proteins of the molecular chaperones that assist in the folding of proteins [32]. The specific endonuclein interacting proteins identified was BiP that is involved in protein translocation, folding, refolding and degradation of proteins in the ER as well as reverse translocation of proteins from the ER to the cytosol for ubiquitination and degradation in the proteasome [26,58]. In presence of ATP, BiP interacts with the J domain of DNAJB11 and may activate the ATPase activity of BiP during its action [29]. Both BiP and DNAJB11 have recently been implied as target genes in the mammalian unfolded protein response (UPR) [59,60]. Endonuclein may thus through its interaction with DNAJB11 and BiP modulate the chaperone activity in the ER.

Finally, two other proteins of the ER were observed to interact with endonuclein. PSBGs which are a group of secretory proteins that are abundantly expressed in the placenta during embryonic development and which may have immunomodulatory functions [44] and SDF2-like protein 1 which is a novel protein that contains three MIR domains. The latter motifs are found in three classes of proteins in the ER [39], protein *O*-mannosyl-transferases, which catalyzes transfer of mannose from dolichyl activated mannose to seryl or threonyl residues of secretory proteins [61] and two classes of intracellular Ca^2+^-release channel proteins, the ryanodine receptors and the inositol 1,4,5-trisphosphate receptors [62]. From these proteins, it is known that the MIR domains in the protein *O*-mannosyl-transferases are essential for its enzymatic function [63] whereas they are involved in binding of inositol 1,4,5-trisphosphate in the signal transduction pathway that releases Ca^2+ ^from intracellular stores. In this respect, it may be of importance that endonuclein can bind two Ca^2+ ^ions, each with a* k*_D _of approximately 240 μM. Endonuclein thereby possesses the ability of binding Ca^2+ ^with the concentrations of free Ca^2+ ^that are present in the ER ranging from about 400 μM in resting cells to below 50 μM after Ca^2+^-mobilization [64]. It is thus possible that the physiologic effect of endonuclein in the ER is Ca^2+ ^dependent. However, the specific functions of the MIR domains in SDF2-like protein 1 must await further studies, although it may be anticipated that the domains presumably are involved in the binding to endonuclein since SDF2-like protein 1 almost exclusively consists of three MIR domains (Fig 4B).

## Conclusions

In conclusion, we have found that the nature of the endonuclein interacting proteins confirms a regulatory role for endonuclein, especially with respect to modulation of signal transduction pathways to the nucleus and chaperone activities in the ER. The specific function(s) of endonuclein in these processes will be the focus of future studies.

## Methods

### Expression of recombinant proteins in Escherichia coli

The coding region (Asn^2^-Ser^501^) of the 2309.2-cDNA [46] was PCR amplified essentially as previously described [65]. The primer used in the sense direction contained a *Bam*HI site (9502F): 5'-CAC-***GGA-TCC***-ATC-GAG-GGT-AGG-AAC-CGC-AGC-CGC-CAG-G-3' and the primer used in the antisense direction contained a *Hind*III site 5'-TTC-***AAG-CTT***-AAG-ACT-CCA-TGG-GTG-TAT-3'. The PCR-amplified DNA-fragment was cut with the appropriate enzymes and finally ligated into the expression vector pT7-PL giving a fusion protein that was synthesized with MGSHHHHHHGSIEGR in the NH_2_-terminal (pT7-PL-2309.2). I.M.A.G.E. Consortium (LLNL) cDNA Clone IDs 743176, 725923 and 704842 in pT7T3D-Pac and 2178588 in pCMV-SPORT6 [66] were obtained from the American Type Culture Collection (Maryland). The clones were sequenced with primer walking directly on the double stranded plasmids with the dideoxynucleotide chain termination technique [67] using deoxyadenosine 5'[α-35S]thiotriphosphate or using the service facility at MWG Biotech AG (Ebersberg, Germany). The sequences of the clones were submitted to the databases under the GenBank accession Nos. GenBank:AF277316, GenBank:AF277317, GenBank:AF277318 and GenBank:AF277319 for the I.M.A.G.E. clone IDs: 2178588 (SDF2-like protein 1/EIP-8), 704842 (DNAJB11/EIP-4), 725923 (TIP-1) and 743176 (mt-SSB). Open reading frames (ORFs) were sequenced on both strands. Outside ORFs we performed mainly one strand sequencing. The coding regions of the TIP-1 cDNA was PCR amplified with specific primers containing specific restriction sites, TIP-1, F: CGT-***GGA-TCC***-ATG-TCC-TAC-ATC-CCG-GGC-CA; TIP-1, R: TCG-***GAA-TTC***-TAG-GAC-AGC-ATG-GAC-TG. The PCR fragment was purified by gel electrophoresis cut with enzymes and ligated into the protein expression plasmid pGEX-4T3 so that the coding region of the protein was made as a fusion protein with glutathion-S transferase, GST according to the manufacturer (GE Healthcare). After subcloning, the plasmid was sequenced to insure that no PCR errors were introduced. Sequence identity search was carried out in the publicly available DNA and protein databases at the National Center for Biotechnology Information, NCBI, using the basic local alignment search tool, BLAST [68]. Sequence analyses were performed with the Gene Inspector™ and the Gene Construction Kit 2™ programs (Textco, Inc, West Lebanon, New Hampshire). The recombinant plasmids were propagated in *E. coli *XL-1 Blue, DH5α or DH10B and sequenced in order to check the cDNA insert for errors introduced during the amplification process. The proteins were expressed and purified from *E. coli *cells essentially as previously described in detail with the pT7-PL vector [69] and according to the manufacturer with pGEX (GE Healthcare). As a control, pure GST protein was purified from cells transformed with the pGEX-4T3 vector containing no insert.

### Affinity purification of endonuclein interacting proteins

Recombinant endonuclein was coupled to cyanogen bromide-activated Sepharose 4B according to the manufacturers description (Pharmacia, Sweden) and packed as a column. A reference column without recombinant endonuclein coupled was similarly packed. A tissue extract from placenta was made as previously described [70]. Identical amounts (several liters) were applied to both columns in MB-buffer (2 mM CaCl_2_, 1 mM MgCl_2_, 140 mM NaCl, 10 mM Hepes, pH 7.4). After washing of the columns in MB-buffer with 1 M NaCl interacting proteins were eluted with 0.1 M glycine buffer (pH 2.7) and neutralized with 1 M Tris-HCl, pH 9. The eluate was concentrated and subjected to two-dimensional gel electrophoresis.

### Two-dimensional gel electrophoresis (2D-PAGE) and silver staining

Two-dimensional gels were run in the first dimension with IPG strips 3-10NL from GE Healthcare. The second dimension was performed on home made polyacrylamide gels (15%T, 0.5%C). Subsequently the gels were silver stained as previously described [71]. After running gels, they were fixed in 50% (v/v) methanol, 12% (v/v) acetic acid, 0.0185% (v/v) formaldehyde overnight or at least for 1 hr, then washed 3 times for 20 min in 35% (v/v) ethanol and pretreated for 1 min in pretreatment solution: 0.02% (w/v) Na_2_S_2_O_3_,5H_2_O, then rinsed in water 2 times for 3 min, stained in 0.2% (w/v) AgNO_3_, 0.028% (v/v) formaldehyde for 20 min, rinsed with water 2 times for 20 s and developed in development solution: 6% (w/v) Na2CO3, 0.0185% (v/v) formaldehyde, 0.0004% (w/v) Na_2_S_2_O_3_,5H_2_O, for approximately 3 min. Finally, development was stopped in stop solution; 50% (v/v) methanol, 12% (v/v) acetic acid.

### Mass spectrometry identification of endonuclein interacting proteins

Silver stained protein spots from gels were excised and kept in 5% (v/v) acetic acid until digestion which was performed at Protana A/S (Odense, Denmark) in a 96-well plate using the protocol essentially as described [72]. Briefly, the gel spots were washed with 50 mM NH_4_HCO_3_/acetonitrile (1:1) followed by dehydration with acetonitrile. The proteins were reduced in 10 mM dithiotreitol (DTT)/50 mM NH_4_HCO_3 _for 1 hour at 56°C, and alkylated in 55 mM iodoacetamide/50 mM NH_4_HCO_3 _at room temp. The gel pieces were washed several times in 50 mM NH_4_HCO_3 _followed by dehydration with acetonitrile. The proteins were digested overnight with trypsin (Promega, modified trypsin) at 37°C and the resulting peptide mixtures were purified on POROS R2 perfusion chromatography material in purification capillaries. The peptide mixtures were eluted with 50% (v/v) methanol, 5% (v/v) formic acid into the nanospray capillary by centrifugation. Peptide sequencing by tandem mass spectrometry (MS/MS) was performed using a nano-electrospray system coupled to a prototype QqTOF (QSTAR) mass spectrometer (Sciex, Toronto, Canada). Each peptide mass and the partial amino acid sequence were arranged in peptide sequence tags [72] and used to search the nrdb database. All the software used for database queries and management of all mass spectrometric data and search results were developed at Protana A/S (Odense, Denmark).

### Analyses of protein interactions by surface plasmon resonance

Protein-protein interactions were studied essentially as previously described [70] in 10 mM Hepes, 150 mM NaCl, 5 mM CaCl_2_, 1 mM EGTA, pH 7.4, 0.005% P20. In short, surface plasmon resonance measurements were performed on a BIAcore 2000 instrument (Biacore, Uppsala, Sweden). This technology relies on the phenomenon of the surface plasmon resonance that occurs when surface plasmon waves are excited at a metal/liquid interface. Light is directed at, and reflected from, the side of the surface not in contact with sample, and surface plasmon resonance causes a reduction in the reflected light intensity at a specific combination of angle and wavelength. Biomolecular binding events cause changes in the refractive index at the surface layer, which are detected as changes in the surface plasmon resonance signal. In general, the refractive index change is proportional to change in mass concentration at the surface layer.

Coupling of endonuclein was performed by using the amine coupling method. Briefly, a continuous flow of HBS buffer (10 mM HEPES pH 7.4, 3.4 mM EDTA, 150 mM NaCl, 0.005% surfactant P20) passing over the sensor surface of a CM5 sensor chip was maintained at 5 μl/min. The carboxylated dextran matrix of the sensor chip flow cell 1 and 2 was activated by the injection of 60 μl of a solution containing 0.2 M N-ethyl-N'-(3-dimethylaminopropyl)carbodiimide and 0.05 M N-hydroxysuccimide in water. Endonuclein was then at a concentration of 50 μg/ml in 10 mM sodium acetate pH 4.0 injected over flow cell 2 with a flow rate of 5 μl/min. Totally 60 μl was injected. The remaining binding sites in both flow cells were blocked by injection of 35 μl 1 M ethanolamine pH 8.5. The surface plasmon resonance signal from immobilized endonuclein generated 3302 BIAcore response units (RU) equivalent to 59 fmol endonuclein/mm^2^. Pregnancy specific β-1 glycoprotein was from Advanced Immunochemical, Inc. (CA). Recombinant hamster BiP/Grp78 was from Calbiochem. TIP-1 was made as GST fusion protein as described above. Proteins were dissolved in 10 mM Hepes, 150 mM NaCl, 5 mM CaCl_2_, 1 mM EGTA, pH 7.4, 0.005% P20 at concentrations of 1 μM or 5 μM and analyzed by injecting 40 μl onto the derivatized sensor chip and the control flow channel. The BIAcore response is expressed in relative response units (RU), *i.e.*, the difference in response between flow cell with immobilized endonuclein and the control flow channel. Injecting two times 20 μl 1.6 M glycine-HCl buffer, pH 3.0 performed regeneration of the sensor chip after each analysis cycle.

### Immunocytochemistry

HaCat keratinocyte cells were grown on coverslips in DMEM supplemented with 10% (vol/vol) fetal bovine serum and antibiotics (penicillin at 100 units/ml and streptomycin at 50 μg/ml). Coverslips were washed in PBS (10 mM phosphate, 150 mM NaCl, pH 7.3), fixed in PBS supplemented with 4% paraformaldehyde, and finally washed in PBS containing 0.1% Triton X-100. The coverslips were incubated with primary antibodies for 45 min, washed in PBS and then incubated with secondary antibodies for 45 min. The primary antibodies were affinity purified rabbit polyclonal anti-endonuclein (previously described in [11]), affinity purified goat polyclonal anti-TIP-1 (Bethyl Laboratories, USA) and affinity purified chicken polyclonal anti-mt-SSB (P16) (Abcam, UK). The secondary antibodies were conjugated with Alexa 488 or Alexa 556, all purchased from Molecular Probes (Invitrogen, Denmark). Labelling was analyzed by confocal laser scanning microscopy (LSM510, Carl Zeiss, Germany). The high specificity of the endonuclein antibody has previously been demonstrated by western immunoblotting [11]. The specificity of the TIP-1 and mt-SSB (p16) antibodies were assessed by ousting the antibodies with the corresponding recombinant protein. In both cases the ousting was complete (not shown).

### Rate dialysis

Ca^2+ ^binding to endonuclein was measured with the protein in solution using rate dialysis with the microchamber procedure essentially as previously described [45]. The experiments were performed in 150 mM KCl, 50 mM Hepes, pH 7.4 at 37°C. Buffers and endonuclein solutions were passed through a Chelex 100 column (BioRad) in order to remove cations before use. Corresponding sets of free Ca^2+ ^concentrations, [Ca^2+^]_free_, and the average number of Ca^2+ ^ions bound to endonuclein, *r*, were measured and fitted to the Scatchard equation by linear regression:

where *k*_D _is the dissociation constant and *n *is the number of binding sites.

## Abbreviations

2D-PAGE: two-dimensional polyacrylamide gel electrophoresis; BiP: immunoglobulin heavy chain binding protein; EIP: endonuclein interaction protein; ER: endoplasmic reticulum; GST: glutathion S-transferase; DNAJB: DnaJ homolog subfamily B; IL: interleukin; mt-SSB: mitochondrial single-stranded DNA binding protein; ORF: open reading frame; PSBG1: pregnancy specific β-1 glycoprotein; TIP-1: Tax interaction protein 1.

## Competing interests

The authors declare that they have no competing interests.

## Authors' contributions

ML analyzed and interpreted the data obtained and participated in drafting the manuscript. MØJ conducted the confocal microscopy and analyzed and interpreted the data obtained. HV performed the 2-D gel electrophoresis and rate dialysis experiments. CJ conducted the surface plasmon resonance analysis. BH conceived the study, conducted the DNA sequencing analysis, the expression of recombinant proteins, the affinity purification of proteins, analyzed and interpreted the data obtained and participated in drafting the manuscript. All authors read and approved the manuscript
